# Supplementary material for: The Intrinsic Parameters of the Polyamide Nanofilm in Thin-Film Composite Reverse Osmosis (TFC-RO) Membranes: The Impact of Monomer Concentration
Source: Membranes (Basel). 2022 Apr 11;12(4):417. doi: 10.3390/membranes12040417 (PMC9032585; doi:10.3390/membranes12040417)
Supplement: Supplementary file 1 [file membranes-12-00417-s001.zip › membranes-1657934-supplementary.pdf]

Supporting information

Membrane preparation

Firstly, the PSF supporting layer was immersed into deionized water for 12 hours before being mounted in a 27 cm × 22 cm Teflon frame for interfacial polymerization. Then the aqueous phase containing a different concentration of monomer MPD, 4% CSA and 2.5% TEA was poured onto the top surface of PSF for 2 min. Next, an N_2 air knife was used to remove the small liquid drops while pouring out the aqueous solution. Subsequently, the TMC solution was poured onto the PSF substrate soaked with MPD solution and kept for 30 s. Afterward, the TMC organic solution was removed and the prepared membrane was vertically placed in the fume hood. Then it was transferred into the oven at 95 ℃ for 8 min for further drying and cross-linking reaction when there are no evident and fast liquid drops flowing down. Finally, the prepared membrane was washed thoroughly with deionized water and stored in deionized water at room temperature for further performance test and characterizations.

Characterization methods

Field emission scanning electron microscope (FE-SEM, Hitachi, SU8010, Japan) was used to characterize the top and back surface of the prepared polyamide membrane, however, it was necessary to coat the PA layer with Pt for 45 s due to its nonconductive property. Besides, as for the characterization of the back surface of membrane, DMF was used to dissolve the interlayer PSF to peel off the polyamide membrane, and they were carefully transferred to the top surface of silicon wafer.

Transmission electron microscopy (TEM, acceleration voltage of 120 kV, Hitachi, 7700, Japan) was used to reveal the information of PA layer cross-section, including its apparent thickness, hollow void size and intrinsic thickness. Before characterization, the Spurr® resin was used to embedding to solidify PA layer, then all of which were cut into ~80 nm ultrathin slices for observation. More than five positions in each membrane were selected to characterize to minimize the subjective random error.

Atomic force microscopy (AFM, Dimension, Icon, Bruker, Germany) was used to obtain the roughness level of prepared polyamide membrane.

Attenuated total reflection flourier transformed infrared spectroscopy (ATR-FTIR, is50 Nicolet) was used to characterize the functional groups of polyamide membrane; the characteristic peak intensity (such as, C = O, C—N, N—H) may indicate the extent of chemical reaction; Chemical composition of the PA surface (700 × 300 μm^2) was determined by X-ray photoelectron spectroscopy (XPS, Kratos AXIS Ultra DLD) with Al Kα (1486.6 eV) as the radiation source. Based on the element content detected, it was helpful to analysis the degree of membrane cross-linking.

Discussion

The thickness of collapsed nodules outline ($\delta_{c\text{-}n}$) is determined by 15 random positions and TFC-9 is used as an example.

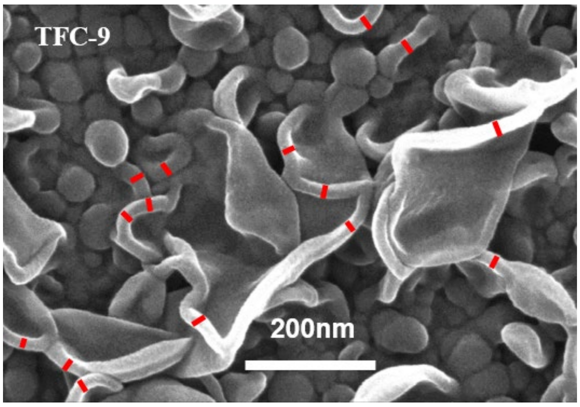

Figure S1. The detailed measurement of $\delta_{c\text{-}n}$ in PA′ top surface based on SEM images (as marked by red line).

The intrinsic thickness (δ_{int}) of PA layer is measured by 15 random locations as Figure S2 shows, herein, TFC-6 prepared by 8.8% MPD & 0.11% TMC is used as an example.

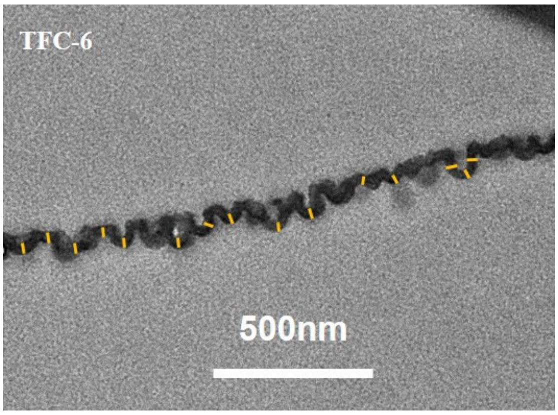

Figure S2. The detailed measurement of δ_{int} based on PA's cross-section parts of TEM images (as marked by yellow line).

The apparent thickness (δ_{app}) of PA layer is measured by 15 random locations as Figure S3 clearly shows, herein, TFC-16 prepared by 8.8% MPD & 0.44% TMC is used as an example.

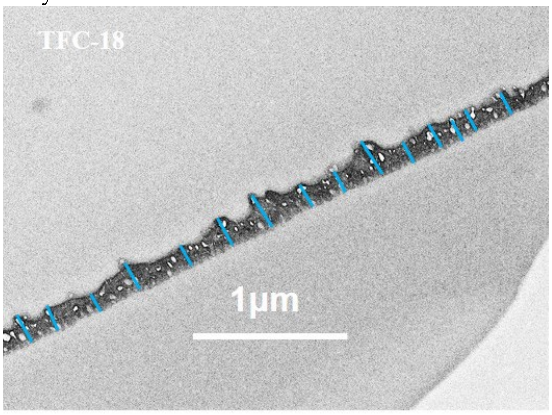

Figure S3. The detailed measurement of δ_{app} based on PA's cross-section parts of TEM images (as marked by blue line).

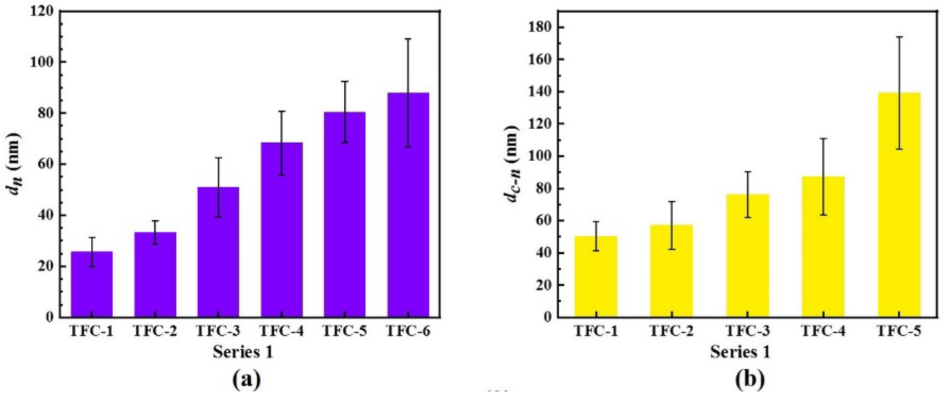

Figure S4. The variation trend of the d_n and the d_{c-n}.in series 1

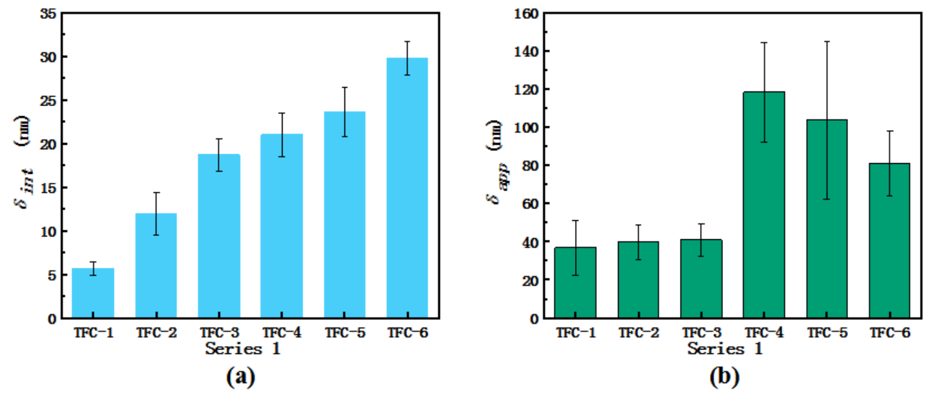

Figure S5. The statistics of the δ_{int} and δ_{app} in series 1

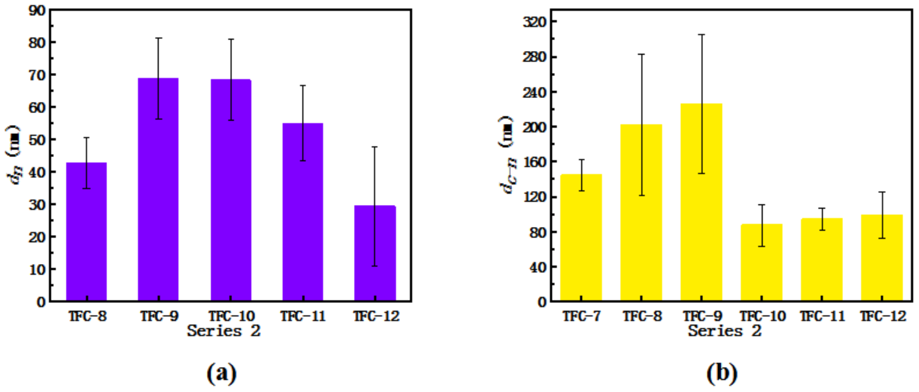

Figure S6. The variation trend of the d_n and $d_{c\text{-}n}$ in series 2

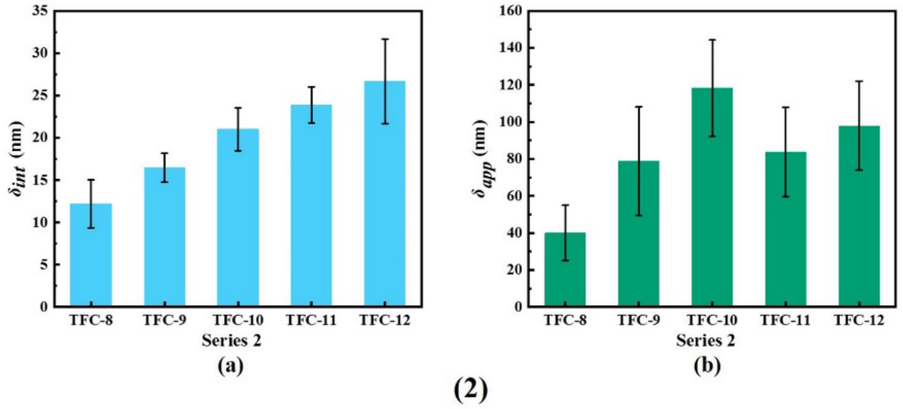

Figure S7. The statistics of the δ_{int} and δ_{app} in series 2

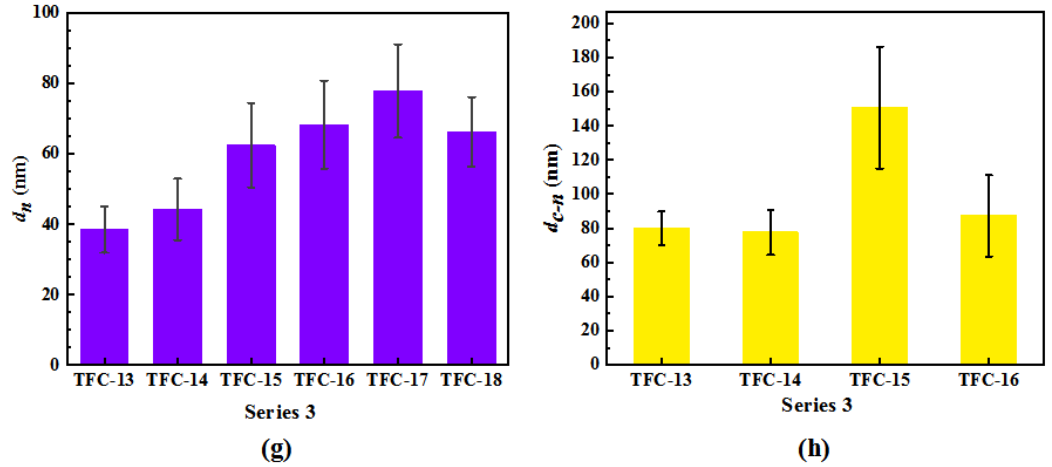

Figure S8. The variation trend of the d_n and d_{c-n} in series 3

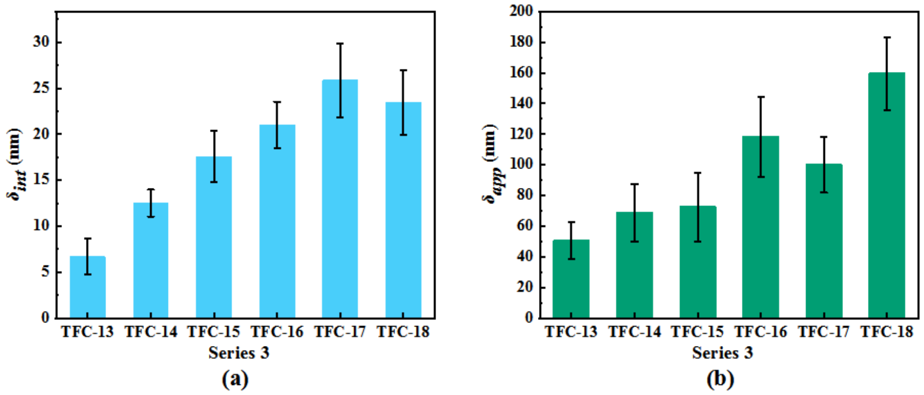

Figure S9. The statistics of the δ_{int} and δ_{app} in series 3

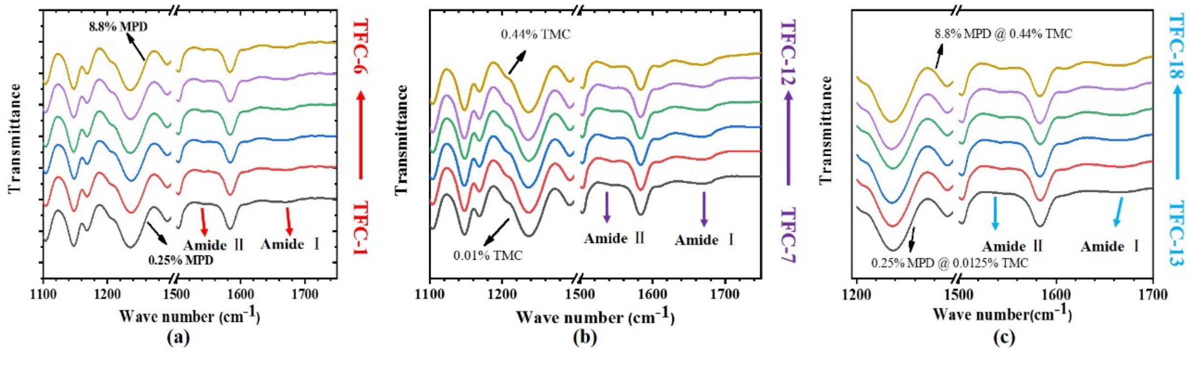

Figure S10. FTIR spectra of TFC membranes: (a) series 1; (b) series 2; (c) series 3.

The variations about the roughness (R_a) of PA's top surface from TFC-1 to TFC-6 in series 1 are shown in Figure S11. And they follow the order of TFC-6 (32.52 ± 8.19 nm) > TFC-5 (24.70 ± 0.72 nm) > TFC-4 (23.60 ± 1.25 nm) > TFC-3 (20.30 ± 0.68 nm) > TFC-2 (19.70 ± 0.73 nm) > TFC-1 (19.78 ± 0.95 nm), which are embodied by that the size of the nodules are enlarging with the increasing of c(MPD).

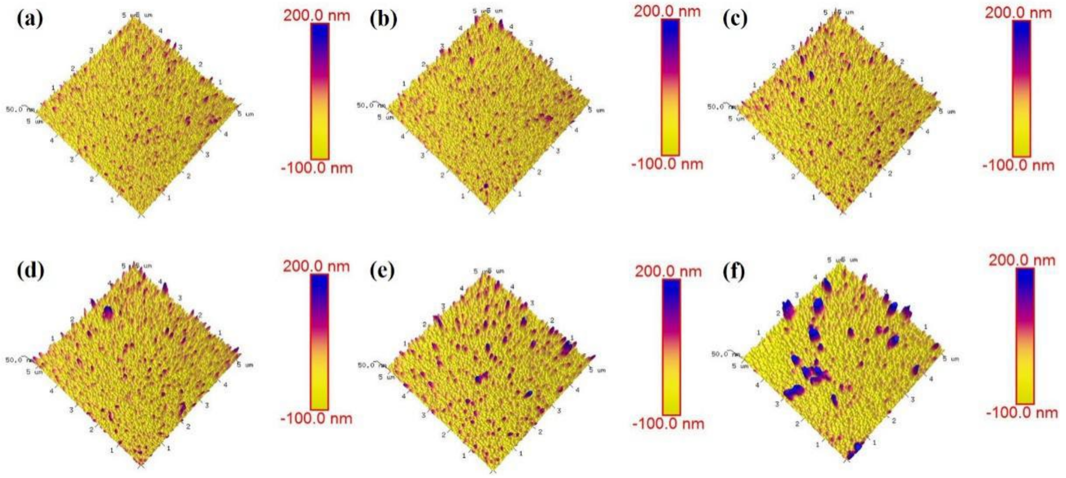

Figure S11. The roughness (R_a) of PA's top surface in series 1: a) ~ f): TFC-1 ~ TFC-6.

The variations about the roughness (R_a) of PA's top surface from TFC-7 to TFC-12 in series 2 are showed in Figure S12. According to the statistics: TFC-9 (61.62 ± 2.07 nm) > TFC-8 (60.02 ± 3.60 nm) > TFC-11 (30.24 ± 2.21 nm) > TFC-12 (27.04 ± 1.31 nm) > TFC-10 (23.60 ± 1.25 nm) > TFC-7 (16.64 ± 0.88 nm). The mountains of big leaf-like or donut-like features lie down the supporting layer PSF due to the such small δ_{int} and could not sustain their initial nodules, which could account for the lowest R_a about TFC-7 in series 2 [1, 2]. The reason may be that the IP reaction will be pushed into the water-oil phase in the circumstances of high *c*(TMC) as TMC is regarded as an inhibitor [3, 4] why TFC-11 prepared by the highest *c*(TMC) doesn't possess the highest R_a.

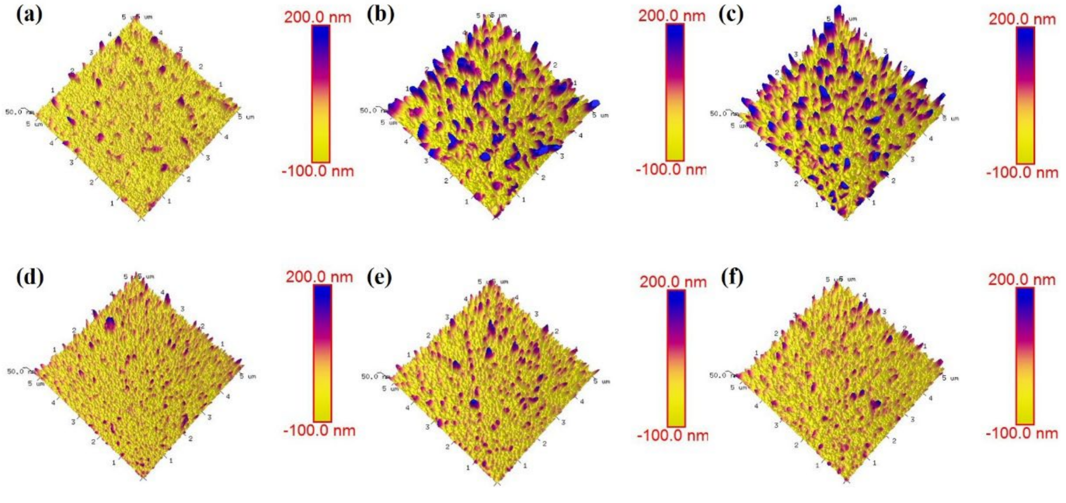

Figure S12. The roughness (R_a) of PA's top surface in series 2: a) ~ f): TFC-7 ~ TFC-12

The variations about the roughness (R_a) of PA's top surface from TFC-13 to TFC-18 in series 2 are showed in Figure S13. According to the statistics: TFC-18 (45.02 ± 4.83 nm) > TFC-15 (42.38 ± 5.82 nm) >TFC-17 (27.06 ± 1.48 nm) > TFC-16 (23.60 ± 1.25 nm) > TFC-14 (22.20 ± 0.20 nm) > TFC-13 (21.86 ± 1.12 nm). Although TMC could function as an inhibitor[3, 4], with the continuous supplement of *c*(MPD) providing the violent driving releasing of nanobubbles, which could still well sharp the PA's morphology, so the highest R_a of all series 3 is TFC-18 prepared by the highest monomer concentration.

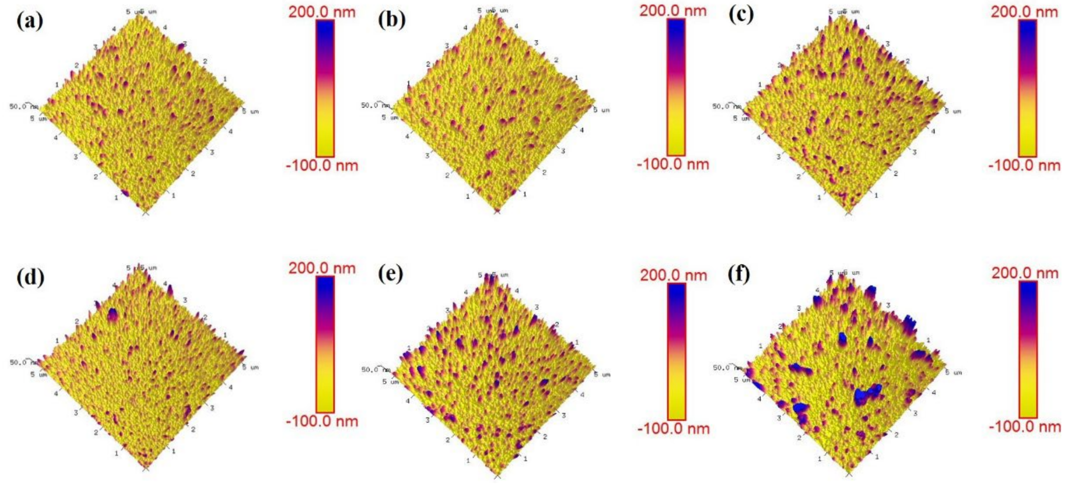

Figure S13. The roughness (R_a) of PA's top surface in series 3: a) ~ f): TFC-13 ~ TFC-18

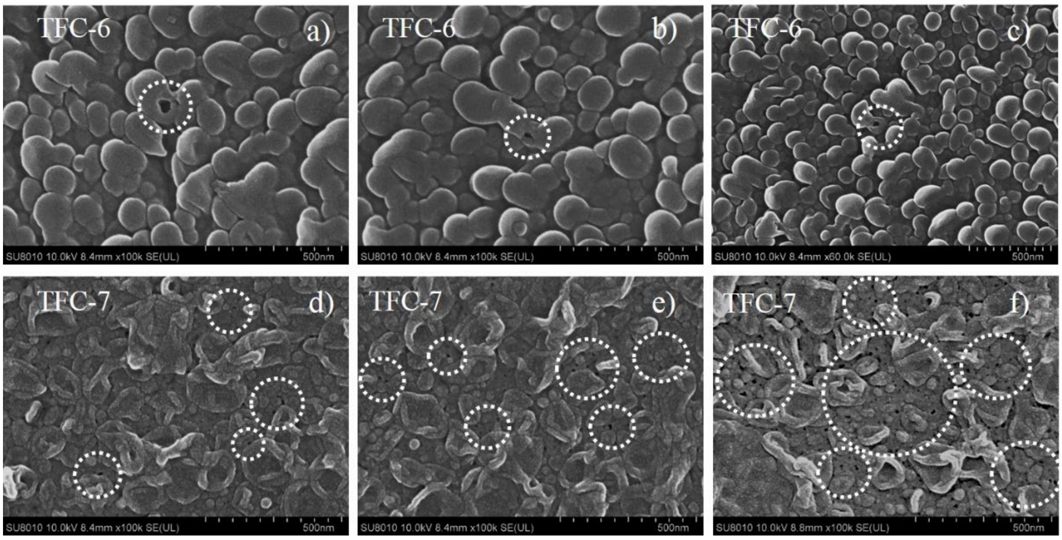

Figure S14. SEM images of the defects on PA's top surface: a) ~ c): TFC-6; d) ~ f): TFC-7.

The defects on topside of PA layer generate where 0.02% surfactant are added into the organic phase to participate with the reaction between monomer MPD and TMC in Fig S15.

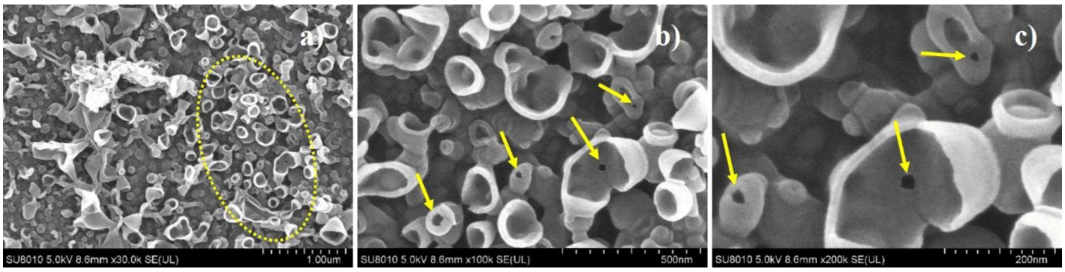

Figure S15. SEM images of PA's defects in the extreme circumstances of membrane preparation.

Table S1. Elemental ratio of PA membrane network.

Series	NO.	O/N
**#1**	TFC-2	1.26 ± 0.06
	TFC-4	1.23 ± 0.02
	TFC-6	1.02 ± 0.07
**#2**	TFC-8	1.03 ± 0.03
	TFC-10	1.23 ± 0.02
	TFC-12	1.69 ± 0.07
**#3**	TFC-14	1.17 ± 0.03
	TFC-16	1.23 ± 0.05
	TFC-18	1.58 ± 0.05

Table S2. The prepared membrane performance.

#	NO.	A (L m^{-2} h^{-1} bar^{-1})	brackish water (2 g L^{-1} NaCl)			seawater (32 g L^{-1} NaCl)			
			J (L m^{-2} h^{-1})	R (%)	B_s (L m^{-2} h^{-1})	J (L m^{-2} h^{-1})	R (%)	B_s (L m^{-2} h^{-1})	R_{born} (%)
1	TFC-1	1.31 ± 0.18	18.46 ± 2.53	98.71 ± 0.70	0.25 ± 0.18	37.29 ± 3.28	96.55 ± 1.02	1.35 ± 0.53	49.45 ± 1.52
	TFC-2	1.94 ± 0.17	26.31 ± 2.45	99.12 ± 0.70	0.23 ± 0.19	44.93 ± 2.86	98.94 ± 0.31	0.48 ± 0.11	62.59 ± 1.46
	TFC-3	2.46 ± 0.04	32.79 ± 0.29	99.50 ± 0.03	0.17 ± 0.01	47.72 ± 0.41	99.40± 0.17	0.29 ± 0.09	69.53 ± 1.53
	TFC-4	3.15 ± 0.02	39.43 ± 1.11	99.69 ± 0.17	0.13 ± 0.07	48.85 ± 1.68	99.64 ± 0.10	0.18 ± 0.05	75.69 ± 0.16
	TFC-5	3.06 ± 0.09	38.10 ± 1.81	99.70 ± 0.06	0.12 ± 0.02	46.15 ± 0.77	99.70 ± 0.04	0.14 ± 0.02	80.37 ± 0.77
	TFC-6	2.74 ± 0.10	33.71 ± 0.60	87.80 ± 0.18	4.70 ± 0.90	43.31 ± 1.07	81.32 ± 0.23	9.95 ± 0.40	61.87 ± 1.15
2	TFC-7		/	/	/	/	/	/	/
	TFC-8	7.64 ± 1.27	85.31 ± 0.55	73.10 ± 14.00	33.51 ± 22.61	80.52 ± 3.33	70.93 ± 13.00	35.21 ± 21.75	57.49 ± 12.79
	TFC-9	7.22 ± 0.18	80.10 ± 6.74	99.43 ± 0.40	0.48 ± 0.35	53.20 ± 2.00	99.52 ± 0.28	0.25 ± 0.14	78.81 ± 0.76
	TFC-10	3.15 ± 0.02	39.43 ± 1.11	99.69 ± 0.17	0.12 ± 0.07	48.85 ± 1.68	99.64 ± 0.10	0.18 ± 0.05	75.69 ± 0.16
	TFC-11	3.04 ± 0.07	36.26 ± 0.67	99.55 ± 0.80	0.16 ± 0.02	42.31 ± 8.02	99.65 ± 0.65	0.15 ± 0.01	76.01 ± 0.71
	TFC-12	1.76 ± 0.01	28.25 ± 1.33	98.78 ± 0.04	0.35 ± 0.01	29.28 ± 0.89	98.34 ± 0.31	0.49 ± 0.08	60.59 ± 4.22

Table S2(continuous)

3	TFC-13	17.45 ± 2.07	181.15 ± 20.21	51.25 ± 1.80	173.20 ± 31.45	94.19 ± 6.68	40.52 ± 0.22	138.29 ± 11.05	0.92 ± 0.91

TFC-14	5.44 ± 1.17	58.20 ± 8.56	70.00 ± 11.00	26.85 ± 16.55	116.46 ± 70.11	80.48 ± 6.89	17.89 ± 9.06	41.29 ± 5.37
TFC-15	4.27 ± 0.12	50.91 ± 0.87	98.56 ± 1.40	0.74 ± 0.74	56.13 ± 0.27	98.98 ± 0.32	0.58 ± 0.18	56.89 ± 0.21
TFC-16	3.15 ± 0.02	39.43 ± 1.11	99.69 ± 0.17	0.12 ± 0.07	48.85 ± 1.68	99.64 ± 0.10	0.18 ± 0.05	75.69 ± 0.16
TFC-17	1.93 ± 0.22	23.17 ± 2.69	99.39 ± 0.23	0.15 ± 0.07	32.49 ± 0.19	99.50 ± 0.27	0.16 ± 0.09	61.20 ± 1.54
TFC-18	0.79 ± 0.12	10.46 ± 0.93	97.84 ± 0.82	0.23 ± 0.11	16.31 ± 1.28	99.12 ± 0.50	0.15 ± 0.09	75.84 ± 0.59

the nanoscale defects in different locations on PA’s top surface are explored and the following SEM images shows their detail morphology in Fig S14.

1. Song, X.; Gan, B.; Yang, Z.; Tang, C.Y.; Gao, C. Confined nanobubbles shape the surface roughness structures of thin film composite polyamide desalination membranes. J. Membr. Sci. 2019, 582, 342–349.
2. Song, X.; Gan, B.; Qi, S.; Guo, H.; Tang, C.Y.; Zhou, Y.; Gao, C. Intrinsic Nanoscale Structure of Thin Film Composite Polyamide Membranes: Connectivity, Defects, and Structure-Property Correlation. Environ. Sci. Technol. 2020, 54, 3559–3569. https://doi.org/10.1021/acs.est.9b05892.
3. Freger, V. Kinetics of film formation by interfacial polycondensation. Langmuir 2005, 21, 1884–1894.
4. Freger, V. Nanoscale Heterogeneity of Polyamide Membranes Formed by Interfacial Polymerization. Langmuir 2003, 19, 4791–4797. https://doi.org/10.1021/la020920q.
